# Localization of a Bacterial Group II Intron-Encoded Protein in Eukaryotic Nuclear Splicing-Related Cell Compartments

**DOI:** 10.1371/journal.pone.0084056

**Published:** 2013-12-31

**Authors:** Rafael Nisa-Martínez, Philippe Laporte, José Ignacio Jiménez-Zurdo, Florian Frugier, Martin Crespi, Nicolás Toro

**Affiliations:** 1 Consejo Superior de Investigaciones Científicas, Estación Experimental del Zaidín Grupo de Ecología Genética, Granada, Spain; 2 Centre National de la Recherche Scientifique, Institut des Sciences du Végétal, Gif-sur-Yvette, France; University of Toronto, Canada

## Abstract

Some bacterial group II introns are widely used for genetic engineering in bacteria, because they can be reprogrammed to insert into the desired DNA target sites. There is considerable interest in developing this group II intron gene targeting technology for use in eukaryotes, but nuclear genomes present several obstacles to the use of this approach. The nuclear genomes of eukaryotes do not contain group II introns, but these introns are thought to have been the progenitors of nuclear spliceosomal introns. We investigated the expression and subcellular localization of the bacterial RmInt1 group II intron-encoded protein (IEP) in *Arabidopsis thaliana* protoplasts. Following the expression of translational fusions of the wild-type protein and several mutant variants with EGFP, the full-length IEP was found exclusively in the nucleolus, whereas the maturase domain alone targeted EGFP to nuclear speckles. The distribution of the bacterial RmInt1 IEP in plant cell protoplasts suggests that the compartmentalization of eukaryotic cells into nucleus and cytoplasm does not prevent group II introns from invading the host genome. Furthermore, the trafficking of the IEP between the nucleolus and the speckles upon maturase inactivation is consistent with the hypothesis that the spliceosomal machinery evolved from group II introns.

## Introduction

Self-splicing group II introns are large ribozymes [1] and mobile retroelements initially identified in the mitochondrial and chloroplast genomes of lower eukaryotes and plants, and subsequently found in bacteria and archaea [2]–[4]. Group II introns display structural, functional and mechanistic similarities to eukaryotic pre-mRNA nuclear introns [5]–[9]. Group II introns typically fold into a conserved three-dimensional structure consisting of six distinct double-helical domains, DI to DVI [10]. Most bacterial group II introns have a multifunctional intron-encoded protein (IEP) open reading frame (ORF) within DIV [4]. Group II IEPs have an N-terminal reverse transcriptase (RT) domain homologous to retroviral RT sequences, followed by a putative RNA-binding domain with RNA splicing or maturase activity (domain X). Some group II IEPs also have a C-terminal DNA-binding (D)/DNA endonuclease (En) region [11], [12]. Upon translation, the IEP remains associated with the full-length group II transcript, forming a ribonucleoprotein particle (RNP) that promotes intron retrohoming into intron-less alleles.

The nuclear genomes of eukaryotes do not contain group II introns, but it has nevertheless been suggested that these introns were the progenitors of eukaryotic spliceosomal introns [13] and of some components of the telomerase and LINE-elements [14]. It has been suggested that group II intron invasion triggered compartmentalization of the nucleus and cytoplasm, and development of the nonsense-mediated RNA decay (NMD) and ubiquitin systems in eukaryotes [15]–[18]. The way in which group II introns might have evolved into spliceosomal introns remains a fundamental question in studies of the molecular evolution of eukaryotes. One study [19] on the nuclear expression of a bacterial group II intron (Ll.ltrB) in the yeast *Saccharomyces cerevisiae* showed that the pre-mRNA harboring the intron was spliced predominantly in the cytoplasm and was subject to NMD, but that the group II intron ORF was nevertheless poorly translated. The targeting of the Ll.ltrB IEP (LtrA) to the nucleus in *S. cerevisiae* required tagging with the SV-40 T-antigen nuclear localization signal (NLS). Bacterial group II intron-mediated gene targeting within eukaryotic cells has been achieved by the co-microinjection of Ll.ltrB-derived RNPs and their cognate homing site into *Xenopus* oocytes. The targeting reaction was shown to be Mg^2+^-dependent and impaired by DNA chromatinization [20]. Both the Ll.LtrB intron RNA and the LtrA protein have also been produced in human cells, but this required resynthesis of the LtrA ORF, with changes in codon usage to match that preferred in higher eukaryotes. Furthermore, the addition of a NLS was required to target LtrA to the nucleus [21]. It therefore remains unclear whether a wild-type group II IEP could alone, or together with the intron RNA, overcome the barrier presented by the nuclear envelope. We investigated the production and intracellular distribution, in bacteria and in *Arabidopsis thaliana* protoplasts, of the *Sinorhizobium meliloti* RmInt1 IEP, which, unlike LtrA, has no endonuclease domain [22]. We generated constructs encoding N- and C-terminal fusions of the IEP to enhanced green fluorescent protein (EGFP). We found that the wild-type fusion protein was uniformly distributed within the cytoplasm of most bacterial cells. By contrast, in *A. thaliana* protoplasts, the full-length IEP was found exclusively in the nucleolus, whereas the maturase domain alone targeted EGFP to nuclear speckles. Our data indicate that the localization of group II introns to the nucleus does not constitute a barrier to the potential extension of group II intron technology to genome engineering in eukaryotes, and provide further support for the hypothesis that group II introns were the precursors of the eukaryotic spliceosomal complex.

## Materials and Methods

### Bacterial Strains and Growth Conditions

*E. coli* strain DH5α was routinely used for the cloning and propagation of plasmid constructs. Exceptionally, pK7WGF2 and pK7FWG2 were maintained in strain DB3.1. Both strains were routinely grown overnight at 37°C on Luria-Bertani (LB) medium. For localization experiments, DH5α was grown overnight at 30°C. *S. meliloti* strain RMO17 (RmInt1 intron-less strain; [23]) was used for homing assays. It was grown on complete trypton-yeast extract medium (TY) or defined minimal medium (MM) [24]. Media were supplemented, when required, with antibiotics, at the following concentrations: kanamycin, 200 µg/ml for *S. meliloti* and 50 µg/ml for *E. coli*; tetracycline, 10 µg/ml; ampicillin, 200 µg/ml and spectinomycin, 100 µg/ml.

### Plasmid Constructs

The genetic constructs transiently expressed for the production of RmInt1 IEP-enhanced green fluorescent protein (EGFP) fusions were obtained with Gateway® technology (Invitrogen). Entry vectors were first generated by the recombination of PCR-amplified IEP-coding DNA fragments with pDONR™ 221 (Invitrogen). The primers used for PCR are listed in table S1. The mutant (NLSm) with a modified putative nuclear localization signal (RPRR→APAA; amino acids 291 to 294 of the IEP) was obtained by site-directed mutagenesis with the Altered Sites II *in vitro* Mutagenesis pAlter-1 System (Promega). The final expression vectors were obtained by recombination of the various donor plasmids with pK7WGF2 (to generate N-terminal fusions to EGFP) and pK7FWG2 (to generate C-terminal fusions) [25], except for pK7-n4, which was obtained by inserting a *Sac*I intron ΔORF fragment from pKGEMA4 (plasmid containing an intron ΔORF derivative; [26]) downstream from the IEP, in the *Sac*I site of pK7-nIEPsac. Intron donor plasmids for *in vivo* homing assays (pKG4cGFP and pKG4nGFP) were constructed with the pK7-nIEP and pK7-cIEP plasmids described above, and the pBBR-MCS2 derivatives pKG0 (intron-less plasmid; [22]) and pKGEMA4. The pKG4cGFP plasmid was obtained as follows. First, pKGEMA4 was restricted with *Spe*I and *Sac*II and the resulting fragment (coding sequence for amino acids 1 to 330 of the IEP) was inserted into pKG0, yielding pKGIE. A *Sac*II fragment from pK7-cIEP (coding sequence corresponding to amino acids 331 to 419 of the IEP, with EGFP fused to the C-terminus) was obtained and inserted into the *Sac*II site of pKGIE, yielding pKGcIEP. A *Sac*I fragment from pKGEMA4 encoding the ΔORF derivative of RmInt1 was inserted into the *Sac*I site downstream from the IEP:EGFP fusion in pKGcIEP, to yield the final construct, pKG4cGFP. The other intron donor plasmid, pKG4-nGFP, was obtained by replacing the wild-type IEP sequence with the N-terminal IEP-EGFP fusion. This fragment was obtained as a *Spe*I-*Eco*RI fragment from pK7-nIEP.

### *In Vivo* Homing and Splicing Assays

**Figure 1:**
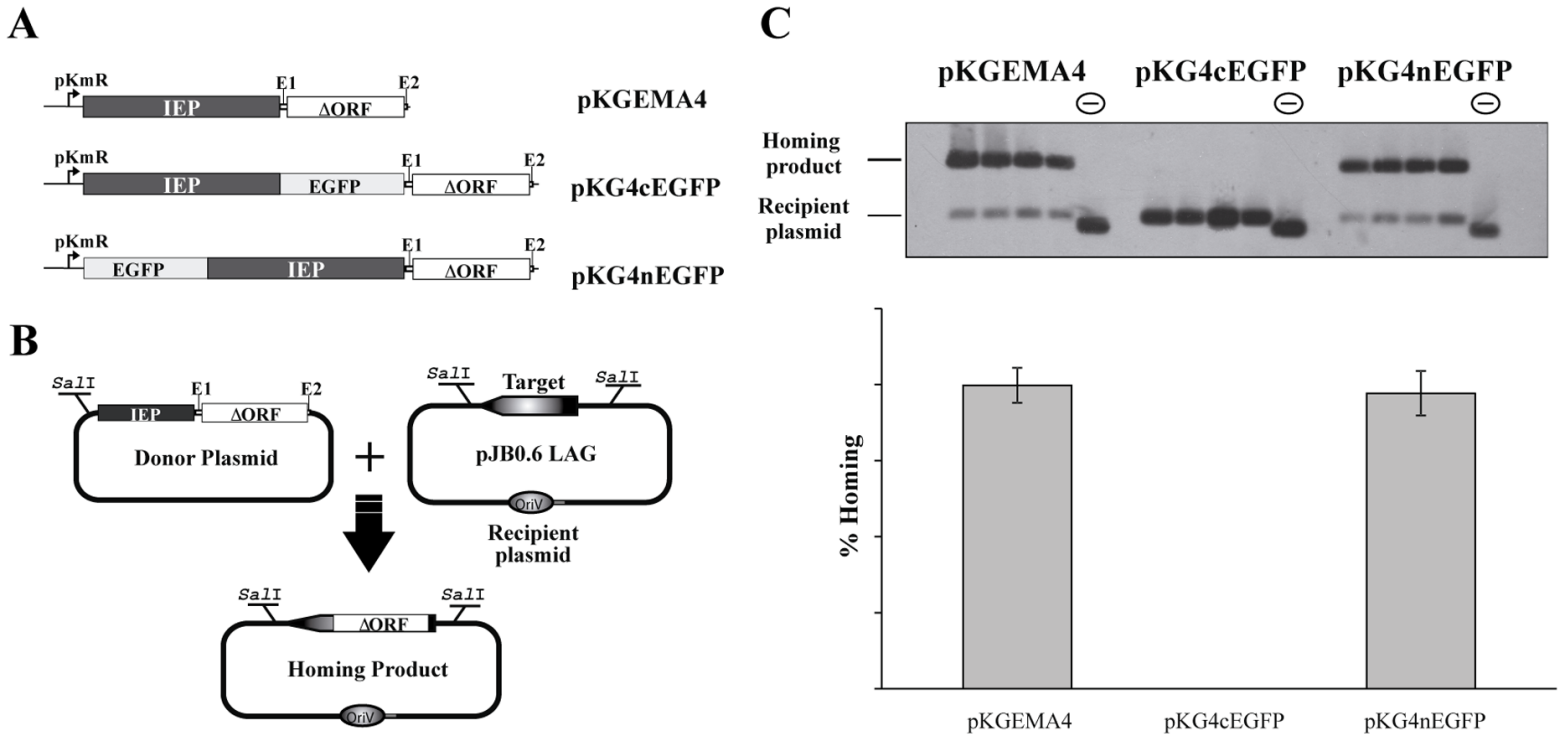
Mobility assays for the IEP-EGFP fusions. (*A*) Schematic diagram of the constructs used in the retrohoming analysis of the WT (pKGEMA4), C-terminal (pKG4cGFP) and N-terminal (pKG4nGFP) fusions; pKm^R^, promoter of the kanamycin resistance gene; IEP, intron-encoded protein of RmInt1; ΔORF; RmInt1 derivative in which domain IV of the ribozyme has been deleted between positions 611 and 1759; EGFP, enhanced green fluorescent protein. (*B*) Outline of the double-plasmid retrohoming assay: the target on the recipient plasmid could be invaded by an intron from the donor, resulting in the homing product. (*C*) Effect of the tagged IEPs on intron homing. For homing assays, plasmid pools from *S. meliloti* RMO17 cells harboring donor (pKGEMA4, pKG4cGFP or pKG4nGFP) and recipient plasmids (pJB0.6LAG) were analyzed by Southern hybridization with a DNA probe specific to the target (insertion sequence IS*Rm2011-2*). The recipient plasmid pJBΔ129 was used as a negative control in the assays (indicated as a minus sign in a circle above the blot). Target invasion rates in each homing assay were calculated as described in Materials and Methods and are plotted in the histogram shown below the blot.

The *in vivo* homing efficiency of the RmInt1 ΔORF-derivatives expressed from pKGEMA4, pKG4nEGFP, pKG4cEGFP and pKG4-NLSm was determined by a double-plasmid assay in *S. meliloti* strain RMO17, using pJB0.6LAG [27] as the recipient plasmid (Figure 1B). Retrohoming events were then detected by the Southern hybridization of *Sal*I-digested plasmid DNA with a digoxigenin (DIG)-labeled probe specific for IS*Rm*2011-2, as previously described [26]. Homing efficiency was calculated as the ratio of homing product to homing product plus non invaded recipient plasmid, converted into a percentage. We determined mean values for at least four independent determinations [28], [29]. Recipient plasmid pJBΔ129, which lacks the RmInt1 target, was used as a negative control in the assays [22].

*In vivo* splicing of the IEP (NLSm)-ΔORF derivative was assessed by primer extension analysis on total RNA from *S. meliloti* RMO17 cells expressing the intron from plasmid pKG4-NLSm, as previously described ([30]; Figure S1A). We used pKGEMA4 as a positive control and pKG4dV (a mutant defective in splicing, in which the catalytic triad GTT is replaced by CGA in domain V of the RmInt1 ribozyme; [26]) as a negative control. The cDNA bands corresponding to the resolved extension products were quantified with the Quantity One software package (Bio-Rad Laboratories). Intron excision efficiency was calculated on the basis of the intensity of the 97 nt product and was normalized against WT levels. Splicing of the ΔC29, YYAA, YAHH and KA IEP mutant - ΔORF derivatives in RMO17 was analyzed in previous studies [28], [31], [32].

### Plant Material and Growth Conditions

*Arabidopsis thaliana* cells (Columbia T87 ecotype) were maintained in solution culture in Jouannean and Peaud-Lenoel medium (JPL) (modified from [33]) containing 0.03 mM NaH_2_PO_4_, 0.37 mM KH_2_PO_4_, 0.06 mM Na_2_HPO_4_, 19.4 mM KNO_3_, 0.45 mM MgSO_4_, 0.9 mM CaCl_2_, 30 µM H_3_BO_3_, 1.5 µM KI, 30 µM MnSO_4_, 90 µM ZnSO_4_, 0.3 µM Na_2_MoO_4_, 0.03 µM CuSO_4_, 0.03 µM CoCl_2_, 5 µM FeSO_4_, 5 µM Na_2_-EDTA, 4.1 µM nicotinic acid, 2.4 µM pyridoxine HCl, 1.2 µM thiamine HCl, 550 µM myo-inositol, 26.6 µM glycine, 0.01% (w/v) casein hydrolysate, 1.5% (w/v) sucrose and 1 µM 2-naphthalene acetic acid, with the pH adjusted to 5.8 with KOH, sterilized by autoclaving. The culture was maintained by weekly 1∶10 dilution with 100 ml of fresh JPL medium. The cells were cultured at 23°C, with constant shaking (120 rpm) and constant low-intensity light (ca. 50· µE m^−2^·s^−1^).

### Isolation and Transfection of Protoplasts

For the isolation of protoplasts, a one-week old *Arabidopsis* cell culture was diluted 1 in 3 [v/v] with fresh JPL medium and cultured as described above for three days. Upon subculture, cells were centrifuged (300×*g* for 5 min) and resuspended in 25 ml of enzymatic solution (1% [w/v] cellulase “Onozuka” R10 and 0.2% [w/v] macerozyme R10 from SERVA Electrophoresis GmbH in JPL-GM medium (JPL medium in which sucrose is replaced with glucose and mannitol, at a final concentration of 30.5 g l^−1^ for both) and then incubated in a final volume of 75 ml JPL-GM for 3.5 hours on a rotatory shaker, under constant light. Digested cells were pelleted by centrifugation (90×*g* for 5 min), washed with JPL-GM, and resuspended in 9.5 ml of JPL medium containing 0.28 M sucrose. Floating protoplasts were harvested by centrifugation (90×*g* for 5 min).

For transient expression assays, 2–4×10^5^ protoplasts (50 µl) were mixed with 15 µg of plasmid DNA (15 µl) and three volumes of PEG solution (25% [w/v] PEG 6000, 450 mM mannitol, 100 mM Ca(NO_3_)_2_). After incubation in the dark for 20 minutes, the protoplasts were washed with 275 mM Ca(NO_3_)_2_ and centrifuged (90×*g* for 5 min). The pellet was resuspended in 500 µl of JPL-GM and incubated in the dark for 18 h before observation under a light microscope.

### Subcellular Localization of IEP-EGFP Proteins

Plasmids expressing the various IEP-EGFP fusion proteins and pEGFP as a control were independently introduced into *S. meliloti* RMO17 by conjugation and into *E. coli* DH5α by electroporation. Aliquots of bacteria (5 µl) from exponential (OD_600_ = 0.6) and stationary (OD_600_ = 2.4) phase cultures were dispensed on glass slides. Fluorescence was analyzed with a Leica DMI6000B microscope (Leica Microsystems) fitted with a x100/1.40 oil immersion lens and a GFP filter, and equipped with a Leica DFC300 FX digital camera.

*A. thaliana* ColT87 protoplasts were transformed with plasmids encoding the various IEP-EGFP fusion proteins and observed 18 hours later. Fluorescence was assessed under a Leica DMI6000B microscope, with a x63/1.40 oil immersion objective. Protoplasts cotransformed with the SRp34-RFP construct, encoding a protein localized specifically in speckles [34], and IEP-EGFP fusions were analyzed with a Leica TCS SP confocal laser scanning microscope (Leica Microsystems). The images obtained were processed with ImageJ ([35]; http://imagej.nih.gov/ij/) and Adobe Photoshop CS4 (Adobe Systems Inc.] software.

### Identification of Excised Intron Forms by Reverse Transcription-polymerase Chain Reaction (RT-PCR)

We isolated total RNA from protoplasts transformed with pK7n4 or pK7n4dV (negative control), using the RNeasy Plant Mini Kit (Qiagen), according to the kit manufacturer’s instructions. RT-PCR was performed as previously described ([30]; Figure S2A). First-strand cDNA synthesis was performed with 8 µg of total cellular RNA, 25 pmol of the Ect1-specific primer (5′-CACCTGCTCGGATCTCGTC-3′) and SuperScript II RNase H^−^ reverse transcriptase (Invitrogen), as indicated in the manufacturer’s protocol. An aliquot (1/15) of the reaction was used as a template for PCR, with 15 pmol of the LL primer (5′-GAGGTTCACGCACCGTTCTG-3′), which was designed to be complementary to a sequence 59 nt from the 3′ end of RmInt1, and 15 pmol of the P primer (5′-TGAAAGCCGATCCCGGAG-3′) complementary to a sequence 97 nt from the 5′ end of the intron. The RT-PCR products (25 µl) were resolved by electrophoresis in a 2% agarose gel. The DNA fragments of the expected size were isolated from the gel with the Illustra™ GFX™ PCR DNA and gel band purification kit (GE Healthcare). The DNA obtained was inserted into the pGEMT-Easy vector (Promega), and 140 clones were sequenced.

## Results and Discussion

### Construction of Active IEP-EGFP Fusions

We studied the intracellular distribution of the RmInt1 IEP in both bacterial and eukaryotic cells, by constructing translational N- and C-terminal IEP-EGFP fusions (Figure 1A). We investigated whether the fusion proteins were still able to promote intron mobility, by assessing the retrohoming of intron-donor constructs in double-plasmid mobility assays in *S. meliloti* strain RMO17 (Figure 1B). The intron-donor plasmid pKGEMA4 was used as the reference for wild-type retrohoming efficiency (fraction of recipient targets invaded by the intron). The target-recipient plasmid used in the assays was pJB0.6LAG (Figure1B), in which the RmInt1 target site (−176/+466) is inserted in the orientation of leading-strand synthesis at DNA replication forks. This arrangement favors the preferred RmInt1 retrohoming pathway, which involves reverse splicing of the intron RNA into the DNA target site and subsequent reverse transcription by the IEP, using the nascent DNA lagging-strand as the primer [27]. No homing products were detected in cells producing the C-terminal IEP-EGFP fusion from pKG4cEGFP. By contrast, the N-terminal IEP-EGFP fusion protein generated from pKG4nEGFP had a retrohoming efficiency into pJB0.6LAG of 77.73%, a value similar to that of the intron-donor plasmid pKGEMA4 (79.77%). We, therefore, conclude that the N-terminal fusion of IEP to EGFP does not affect intron mobility. We used this configuration to investigate the distribution of the RmInt1 IEP.

### Localization of IEP-EGFP Fusions in Bacteria

**Figure 2:**
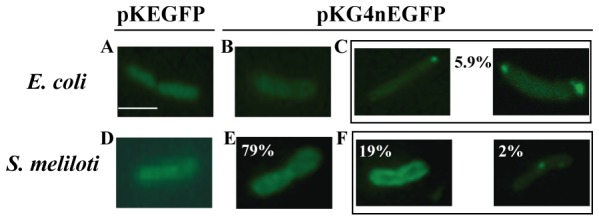
Localization of the N-terminal IEP-EGFP fusion in bacteria by fluorescence microscopy. The constructs used in the assays are indicated above the panels. *E. coli* and *S. meliloti* were grown at 30°C. Panels *A* to *C* correspond to *E. coli* DH5α; Panels *D* to *F* correspond to *S. meliloti* RMO17. The numbers within the panels indicate the percentage of cells displaying the corresponding fluorescence pattern. Scale bar ≈ 2 µm.

It has been reported [36] that LtrA, produced either alone or in complex with the Ll.ltrB RNA in bacteria, localizes to the cellular poles in both *E. coli* and its natural host, *L. lactis*. The *S. meliloti* RmInt1 intron has also been shown to be mobile in heterologous hosts, such *E. coli*. We therefore investigated the distribution of the RmInt1 IEP in *E. coli* DH5α and *S. meliloti* RMO17 cells harboring pKG4nEGFP, which encodes the IEP-EGFP fusion and the intron RNA. Bacteria were grown to the exponential growth and stationary phases in rich broth at 30°C, the temperature at which the fluorescence intensity of the fusion protein was highest. No clearly growth-dependent fluorescence patterns were observed. Figure 2 summarizes observations in bacteria grown to stationary phase. Most of the *E. coli* DH5α cells (94.1%) displayed diffuse fluorescence in our assays, indicating a homogeneous intracellular distribution of the IEP-EGFP fusion protein, similar to that of EGFP produced alone from the control plasmid pKEGFP (Figure 2A and B). In the remaining cells (5.9%), fluorescence accumulated in one or two foci localized at the poles (Figure 2C). In *S. meliloti,* we observed three different intracellular fluorescence patterns: *i*) 79% of the cells displayed diffuse fluorescence (Figure 2E), similar to that of the EGFP control (Figure 2D); *ii*) 19% of the cells displayed fluorescence at the periphery of the cell (Figure 2F left) and *iii*) 2% of the cells had only one focus of fluorescence (Figure 2F right). Thus, the polar intracellular localization of the RmInt1 IEP in *E. coli* differed from that of LtrA in that the percentage of cells displaying diffuse fluorescence was higher. Furthermore, the distribution observed in *S. meliloti* was not the same as that of LtrA in its natural host, possibly reflecting a lack of insertion site preference of RmInt1 in the *S. meliloti* genome.

### Subcellular Localization of IEP-EGFP Fusions in Eukaryotic Cells

**Figure 3:**
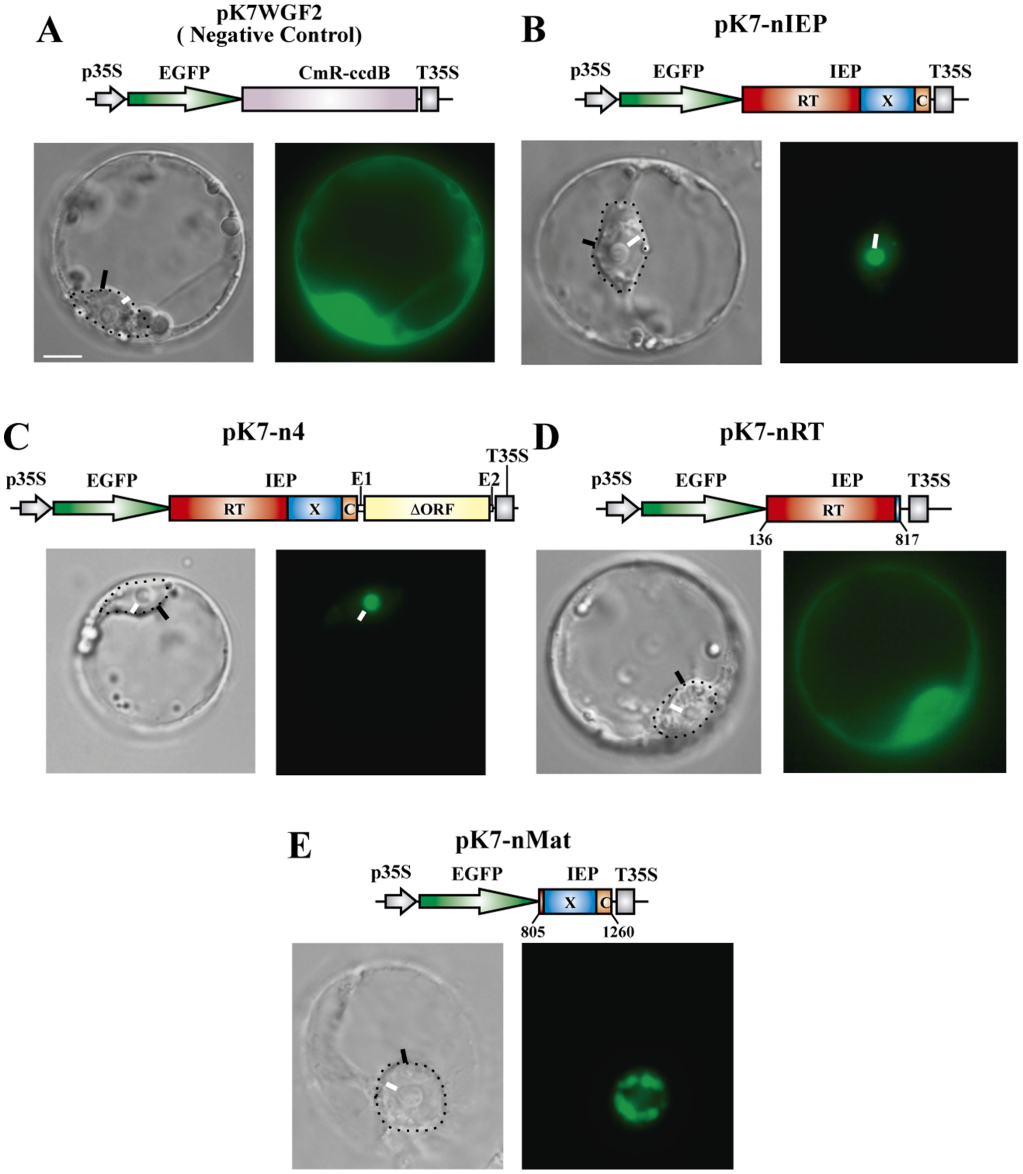
Transient expression and subcellular localization in *A. thaliana* protoplasts of the IEP-EGFP N-terminal fusion. Images show fluorescence and bright-field microscopy. *A. thaliana* protoplasts were transfected as indicated in the Materials and Methods, with the constructs shown over the corresponding panels. The domains of the IEP are represented: in red, the reverse transcriptase; in blue, the maturase; and in orange, the C-terminal domain. The coordinates (nucleotides) of the different IEP subsegments cloned are indicated beneath the diagrams. RmInt1-ΔORF is indicated as a yellow box with flanking short exons E1(−20)/E2(+5). The IEP-EGFP fusion and derivatives were expressed under the control of the CaMV 35S promoter. The negative control used in the experiments was pK7WGF2. On the bright-field images, the nuclei are indicated by a dotted line with a black arrowhead; white arrowheads indicate nucleoli. (Scale bar ≈ 10 µm).

We investigated the intracellular distribution of the RmInt1 IEP in *A. thaliana* protoplasts, using pK7WGF2 [25] as the basis for all constructs for the transient expression of translational fusions of EGFP to the N-terminus of the full-length wild-type IEP and of several mutant and truncated derivatives, under the control of the cauliflower mosaic virus P35S promoter. Protoplasts transformed with the control construct, pK7WGF2, encoding EGFP alone, displayed diffuse fluorescence (Figure 3A), whereas protoplasts transformed with the construct encoding the wild-type IEP fused to EGFP (pK7-nIEP) displayed fluorescence concentrated in the nucleolus (Figure 3B). In other experiments, the IEP was also localized in the nucleolus when coexpressed with the RmInt1 ribozyme from pK7-n4 (Figure 3C). We investigated whether a specific region of the RmInt1 IEP targeted the protein to the nucleolus, by first tagging the maturase and RT segments of the IEP with EGFP. Protoplasts transformed with the construct encoding the EGFP-RT fusion (pK7-nRT) displayed the same pattern of diffuse fluorescence as the control (Figure 3D). By contrast, transformation with the construct encoding the EGFP-maturase fusion (pK7-nMat) resulted in the accumulation of the IEP in nuclear structures resembling speckles (interchromatin granule clusters), but not in the nucleolus (Figure 3E). This suggests that the maturase domain is sufficient to target the RmInt1 IEP to the nucleus, but that a complete functional protein is required for the localization of the protein to the nucleolus.

**Figure 4:**
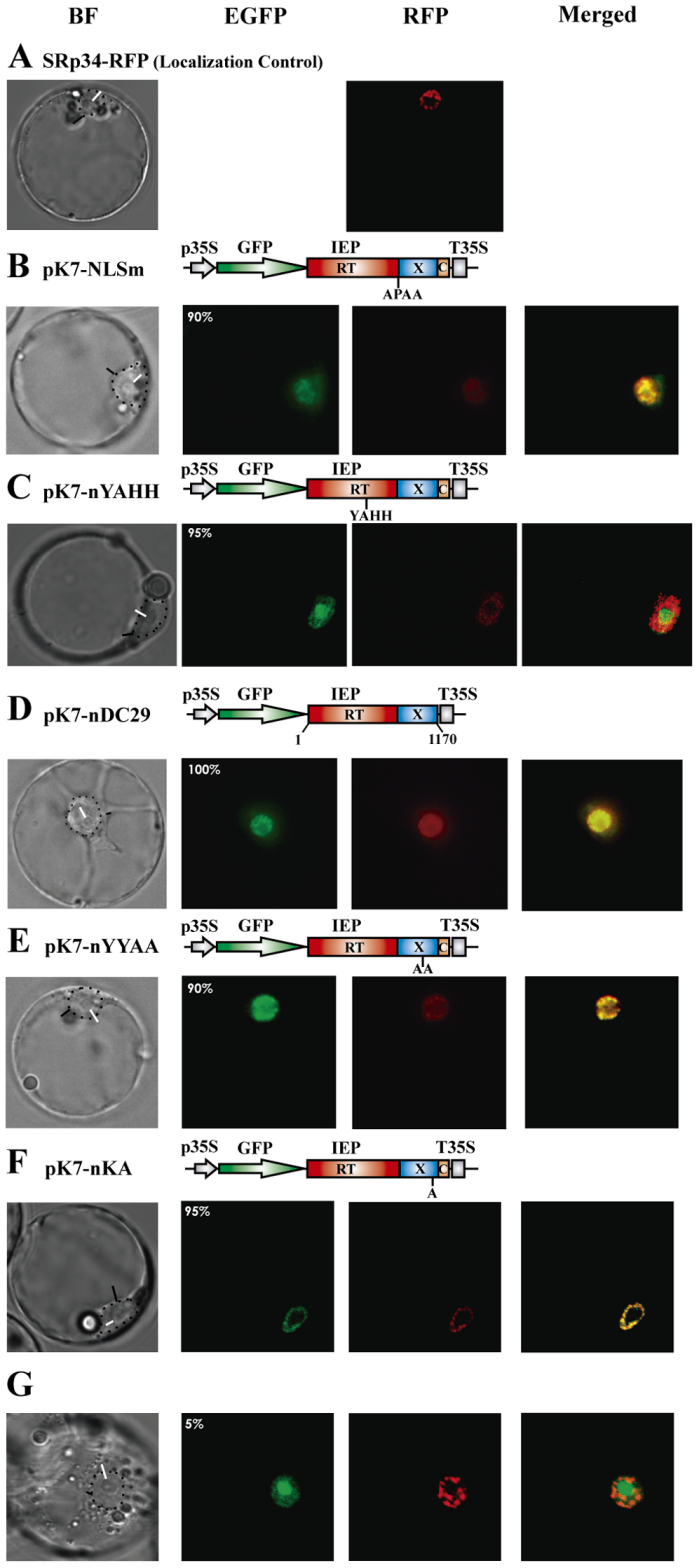
Determination of the localization patterns of various mutant IEP-EGFP fusions in *A. thaliana* protoplasts. Images show fluorescence and bright-field microscopy. *A. thaliana* protoplasts were transfected as indicated in the Materials and Methods, with the constructs indicated above the corresponding panels. The domains of the IEP are represented: in red, the reverse transcriptase; in blue, the maturase; and in orange, the C-terminal domain. For pK7-nΔC29, the coordinates (nucleotides) of the IEP subsegment cloned are indicated beneath the diagram. The wild-type IEP-EGFP fusion and derivatives were expressed under the control of the CaMV 35S promoter. In the colocalization experiments, we used the SRp34/31-RFP construct [26], the product of which localizes to nuclear speckles. On the bright-field images, we indicate the nuclei by a dotted line and a black arrowhead; white arrowheads indicate nucleoli. Panels *C*, *F*, and *G* show confocal microscopy images. Images were merged with ImageJ software. The numbers within the EGFP panels indicate the percentage of transformed protoplasts displaying the corresponding fluorescence pattern. (Scale bar ≈ 10 µm).

PSORT ([37]; http://psort.hgc.jp/form.html) predicted the presence of a “pat4” NLS at the N-terminus of the maturase domain. The putative NLS of RmInt1 IEP consists of three arginine residues and one proline (RPRR) residue in amino-acid positions 291–294 of the protein. We investigated whether this signature was responsible for targeting the RmInt1 IEP to the nucleus, by replacing the arginine residues with alanine residues. This mutation (pK7-NLSm) shifted the fluorescence to the nuclear speckles, outside the nucleolus (Figure 4B), as for the splicing factor SRp34/SR1 fused to RFP [34] (Figure 4A), which was used as the localization marker in these experiments. Thus, these four amino acids do not function as an authentic NLS. Instead, they are required for the nucleolar localization of the RmInt1 IEP. In other experiments, we observed that this mutant IEP could not promote either intron splicing or intron mobility in bacteria (Figure S1A and B, respectively). Thus, the non nucleolar localization of the mutant IEP may result from the misfolding of the protein or a lack of maturase activity.

We then investigated the effect of other IEP mutations affecting either the RT or maturase domain on the distribution of the RmInt1 IEP. In most *A. thaliana* protoplasts (95%) transformed with a construct encoding EGFP fused to an IEP carrying the RT mutation YADD→YAHH, resulting in the retention of 80% wild-type levels of splicing activity [28], the IEP was localized in the nucleolus (Figure 4C). In the remaining 5% of the cells, fluorescence accumulated in nuclear speckles (data not shown). These data indicate that the nucleolar localization signature of the IEP is located in the IEP maturase rather than the RT domain. Consistent with this observation, all the protoplasts transformed with the pK7-nΔC29 plasmid, encoding EGFP fused to an IEP with a truncation of the last 29 C-terminal amino acids of the maturase domain (a mutation that blocks RmInt1 splicing [31]) were colocalized in nuclear speckles with the SRp34 fusion (Figure 4D). Furthermore, a similar colocalization was observed in 90% of the transformed cells expressing the YY→AA (RmInt1 residues 354–355) IEP mutant-EGFP fusion from the pK7-nYYAA construct (Figure 4E). This mutation abolishes intron RNA splicing [31]. Finally, expression of the maturase mutant K381A-EGFP fusion (pK7-nKA; Figure 4F), which displays 30% wild-type levels of splicing in *S. meliloti* [32], resulted in fluorescence located predominantly in the nucleolus (Figure 4G). Together, these results suggest that the localization of the RmInt1 IEP in nuclear speckles is probably dependent on the physical properties and amino-acid composition of the maturase domain, but that nucleolar localization is linked to the maturase activity of the IEP.

### Detection of Spliced Forms of RmInt1 in *A. Thaliana* Protoplasts

We checked whether the ΔORF RmInt1 variant was indeed spliced in protoplasts, by analyzing RNA preparations by RT-PCR, as previously described ([30]; Figure S2A; see Materials and Methods), and resolving the PCR products by electrophoresis in 2% agarose gels (Figure S2A). No amplification products derived from bacteria producing a non functional ribozyme (pK7n4dV) or from mock samples (−RT) were detectable on gels. Conversely, multiple RT-PCR products, putatively derived from RmInt1 splicing, were detected when the wild-type ribozyme was produced from pK7n4 (lanes 1 and 2). Nonetheless, the sizes of these PCR products did not match the expected size for a wild-type intron-spliced lariat. Indeed, the sequencing of up to 140 plasmid inserts obtained by cloning DNA excised from gels revealed that these PCR products were derived from a wide range of processed forms, rather than from the full-length intron lariat (Figure S2B and C). These results indicate that the RmInt1 derivative intron ΔORF is expressed, and probably spliced in protoplasts, but that it is then subject to further modifications.

## Conclusions

It has been suggested [9], [13], [16] that, at an early stage in the evolution of eukaryotes, the ancestral group II intron structure was split into the non-catalytic spliceosomal introns and the catalytically active RNA component of the spliceosome. This transition was accompanied by the degradation of the reverse transcriptase ORF. The evolution of eukaryotic cell organization may also have been a defensive response to the deleterious effect of group II intron proliferation in the host genome [17], [18]. Our data reveal that the compartmentalization of eukaryotic cells into nucleus and cytoplasm does not prevent group II intron invasion of the host genome, but it may control proliferation of the intron, through transient or stable nucleolar sequestration. Strikingly, when the IEP loses its maturase activity, the protein becomes localized in nuclear speckles, domains of the nucleus enriched in pre-mRNA splicing factors [38], including small nuclear ribonucleoproteins (snRNPs) and serine-arginine (SR) proteins, located in the interchromatin regions of the nucleoplasm. Thus, we found that a bacterial group II intron IEP that had lost its maturase activity was colocalized with splicing factors in eukaryotic cells, providing support for the hypothesis that eukaryotic spliceosomal introns may have evolved from group II introns. Land plant mitochondrial and plastid genomes contain various IEPs that are essential for the splicing and spread of organellar introns, but angiosperm nuclear genomes also encode group II intron maturase-related proteins that are localized to the mitochondria. Four maturase genes were initially identified in *Arabidopsis* and *Oryza sativa* [39] and many new nucleus-encoded maturases have recently been identified in other angiosperms, lycophytes and mosses [40]. It has been suggested that they arose by multiple shared and independent transfers of mitochondrial paralogs to the nuclear genome during land plant evolution [40]. Maturases may have persisted in plants, during evolution, by acquiring a targeting signal enabling them to function within the organelles, to support the splicing of organellar group II introns.

Localization of group II intron RNPs to the nucleus, DNA chromatinization and low magnesium concentration impair the use of bacterial group II introns for genomic engineering in eukaryotic cells. We found that unmodified RmInt1 IEP was localized to the nucleus, supporting further investigations of the possible use of this intron for gene targeting in higher organisms.

## Supporting Information

Figure S1 **Effect of the mutation of the putative nuclear localization signal (NLSm; RPRR→APAA; amino acids 291 to 294 of the IEP) on ΔORF splicing and homing** ***in vivo*** **.** (*A*) Primer extension. The analysis was performed on total RNA (20 mg) from RMO17 cells harboring intron-donor plasmids pKG4-NLSm, pKGEMA4 and pKG4dV as a negative control. The 97 nt cDNA product corresponds to the excised intron RNA (S), whereas the larger products, of 110, 126 and 130 nt, are derived from unspliced precursor RNA molecules (Pr). Schematic diagrams of the primer extension products are shown to the right of the panel. (*B*) Homing of the wild type, ΔORF and NLSm mutants. For homing assays, plasmid pools from RMO17 cells harboring donor and recipient (pJB0.6LAG) plasmids were analyzed by Southern hybridization with a DNA probe specific for the insertion sequence IS*Rm2011-2*. The recipient plasmid pJBΔ129 was used as a negative control in the assays. M; DIG-labeled molecular weight markers II and III, from Roche Applied Science.
(TIF)

Figure S2 **Identification of excision products from the RmInt1** Δ**ORF derivative in** ***A. thaliana*** **protoplasts.** (*A*) The products of RmInt1 *in vivo* were detected by reverse transcription and PCR. Reactions were carried out with (+) and without (–) prior reverse transcription (RT) of RNA from *A. thaliana* protoplasts harboring pK7n4 and pK7n4dV as an additional negative control. A schematic representation of the PCR products for the intron lariat and intron circles is also shown, in which the circled A corresponds to the bulged adenosine in domain VI. The products of the RT-PCR are shown on the agarose gel. Dil; dilutions of the cDNA for the subsequent PCR, M; Molecular weight marker. (*B*) Magnification of the sequence from pK7n4. The numbers on the right indicate base positions, taking position +1 to be the first nucleotide of the intron. Numbers within asterisks indicate the omitted intron nucleotides. Numbers with a comma indicate base positions, taking as the new position +1 the next nucleotide behind the 3′ end of the intron. The sequence of the IEP 3′ end is shown in green, the sequences of the spacers between the IEP and the T35S are shown in black, with the intron shown in red. Primers are depicted as horizontal arrows below the corresponding sequence. Primer names are indicated below the arrows. (*C*) Diagram of the different processed forms obtained from sequencing the RT-PCR products. The 5′ and 3′ ends of the intron molecule are indicated. Primers used for amplification indicated as arrowheads below the diagrams. The number in brackets to the left of the diagrams corresponds to the clones found during sequencing. The positions of the intron and exons are indicated by the coordinates above the diagrams. in the colors used are consistent with those in B. Letters in brackets correspond to extra nucleotides found at the junction between the 5′ and 3′ ends of the intron. The vertical line indicates the junction of the 3′ and 5′ ends of the intron.
(TIF)

Table S1 **Primer pairs used to generate the IEP PCR fragments for recombination with pDONR^TM^221.**
(DOCX)
