# Supplementary figures and images for: Localization of a Bacterial Group II Intron-Encoded Protein in Eukaryotic Nuclear Splicing-Related Cell Compartments
Source: PLoS One. 2013 Dec 31;8(12):e84056. doi: 10.1371/journal.pone.0084056 (PMC3877140; doi:10.1371/journal.pone.0084056)

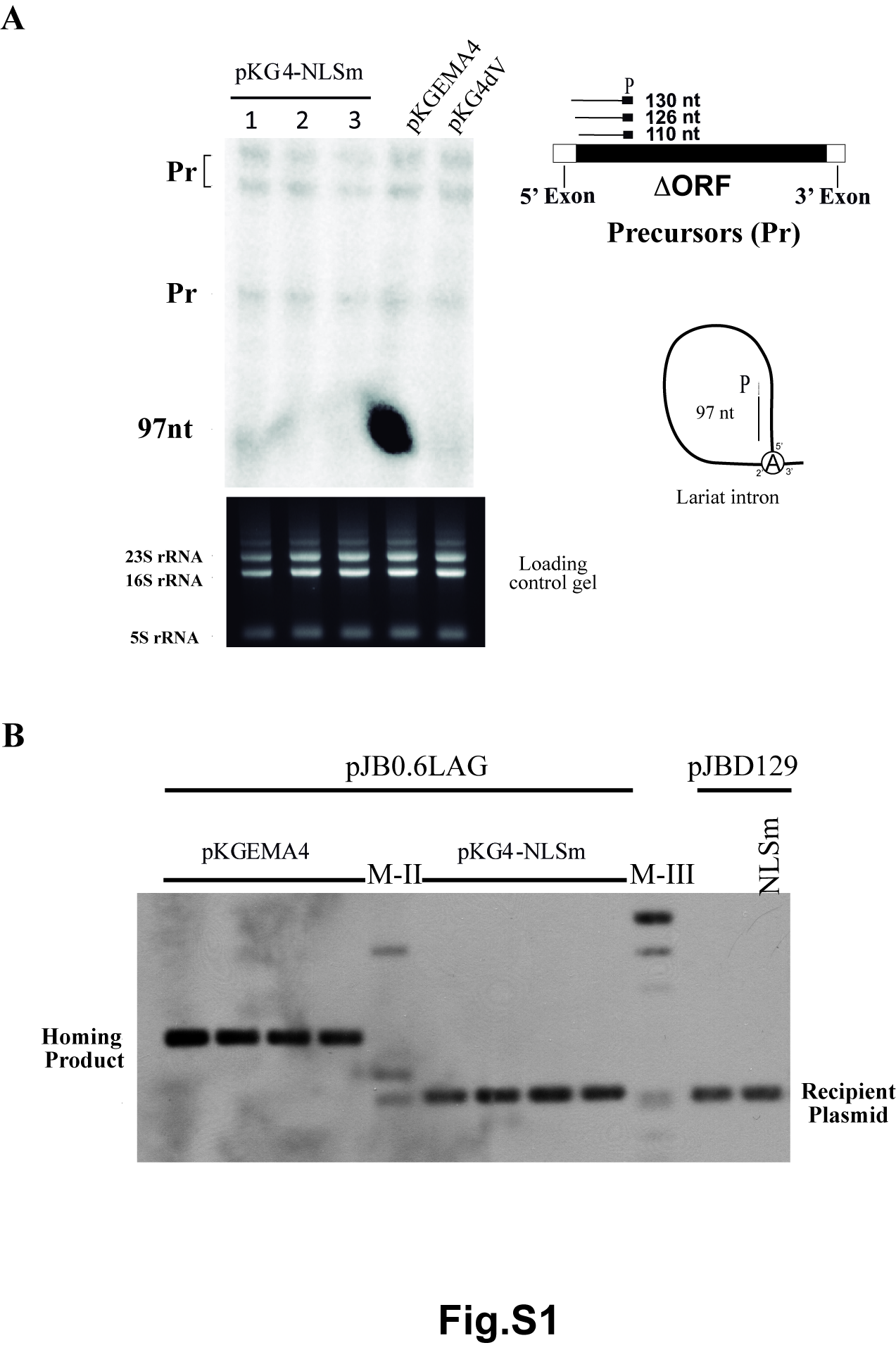

Supplement: Figure S1 — Effect of the mutation of the putative nuclear localization signal (NLSm; RPRR→APAA; amino acids 291 to 294 of the IEP) on ΔORF splicing and homing in vivo . (A) Primer extension. The analysis was performed on total RNA (20 mg) from RMO17 cells harboring intron-donor plasmids pKG4-NLSm, pKGEMA4 and pKG4dV as a negative control. The 97 nt cDNA product corresponds to the excised intron RNA (S), whereas the larger products, of 110, 126 and 130 nt, are derived from unspliced precursor RNA molecules (Pr). Schematic diagrams of the primer extension products are shown to the right of the panel. (B) Homing of the wild type, ΔORF and NLSm mutants. For homing assays, plasmid pools from RMO17 cells harboring donor and recipient (pJB0.6LAG) plasmids were analyzed by Southern hybridization with a DNA probe specific for the insertion sequence ISRm2011-2. The recipient plasmid pJBΔ129 was used as a negative control in the assays. M; DIG-labeled molecular weight markers II and III, from Roche Applied Science. (TIF) [file pone.0084056.s001.tif]

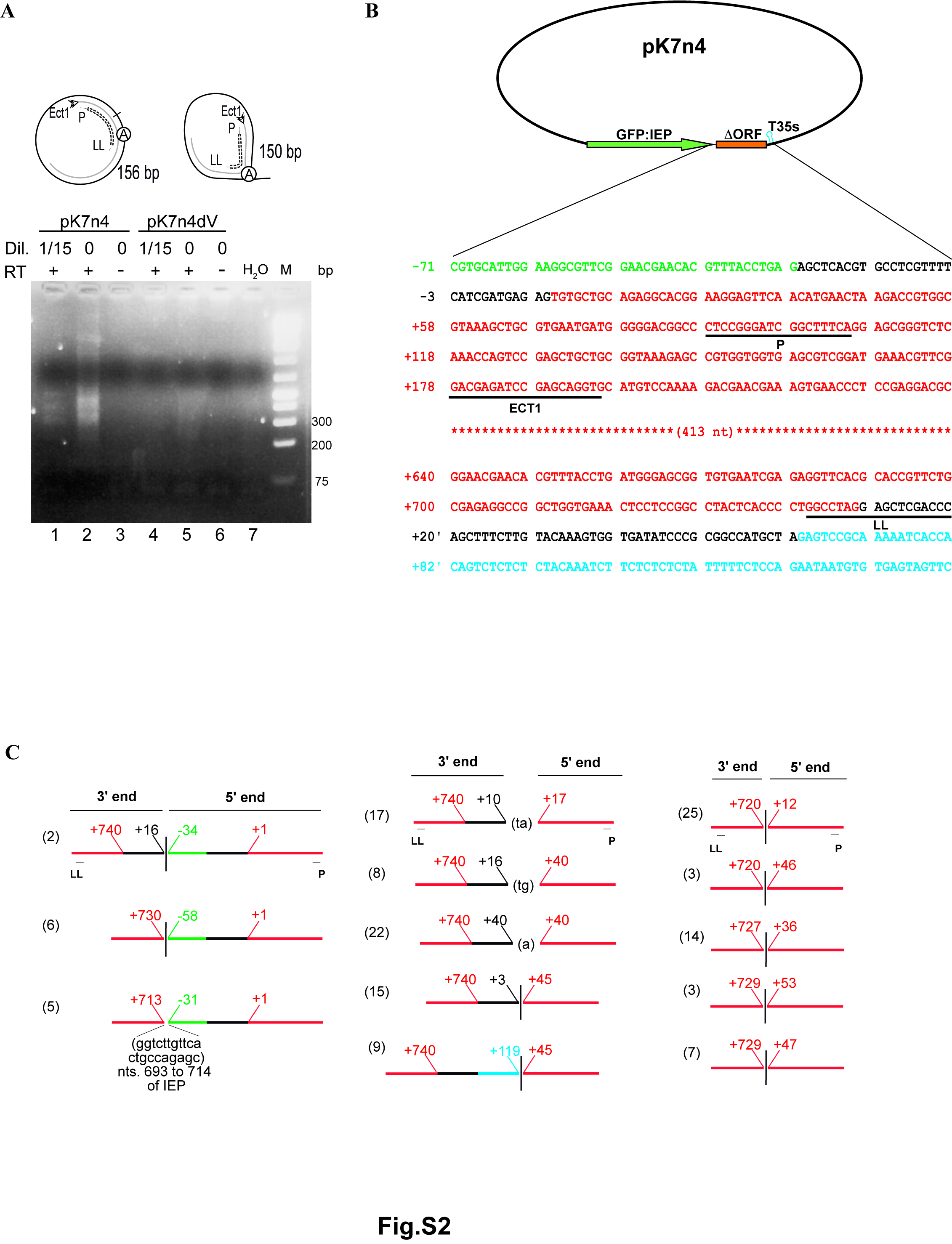

Supplement: Figure S2 — Identification of excision products from the RmInt1 ΔORF derivative in A. thaliana protoplasts. (A) The products of RmInt1 in vivo were detected by reverse transcription and PCR. Reactions were carried out with (+) and without (–) prior reverse transcription (RT) of RNA from A. thaliana protoplasts harboring pK7n4 and pK7n4dV as an additional negative control. A schematic representation of the PCR products for the intron lariat and intron circles is also shown, in which the circled A corresponds to the bulged adenosine in domain VI. The products of the RT-PCR are shown on the agarose gel. Dil; dilutions of the cDNA for the subsequent PCR, M; Molecular weight marker. (B) Magnification of the sequence from pK7n4. The numbers on the right indicate base positions, taking position +1 to be the first nucleotide of the intron. Numbers within asterisks indicate the omitted intron nucleotides. Numbers with a comma indicate base positions, taking as the new position +1 the next nucleotide behind the 3′ end of the intron. The sequence of the IEP 3′ end is shown in green, the sequences of the spacers between the IEP and the T35S are shown in black, with the intron shown in red. Primers are depicted as horizontal arrows below the corresponding sequence. Primer names are indicated below the arrows. (C) Diagram of the different processed forms obtained from sequencing the RT-PCR products. The 5′ and 3′ ends of the intron molecule are indicated. Primers used for amplification indicated as arrowheads below the diagrams. The number in brackets to the left of the diagrams corresponds to the clones found during sequencing. The positions of the intron and exons are indicated by the coordinates above the diagrams. in the colors used are consistent with those in B. Letters in brackets correspond to extra nucleotides found at the junction between the 5′ and 3′ ends of the intron. The vertical line indicates the junction of the 3′ and 5′ ends of the intron. (TIF) [file pone.0084056.s002.tif]
