# Supplementary material for: Localization of a Bacterial Group II Intron-Encoded Protein in Eukaryotic Nuclear Splicing-Related Cell Compartments
Source: PLoS One. 2013 Dec 31;8(12):e84056. doi: 10.1371/journal.pone.0084056 (PMC3877140; doi:10.1371/journal.pone.0084056)
Supplement: Table S1 — Primer pairs used to generate the IEP PCR fragments for recombination with pDONRTM221. (DOCX) [file pone.0084056.s003.docx]

| **Final Constructs** | **Fusion** | **Primer Pairs** | **IEP Positions nts.** | **Primer Sequence** |
| --- | --- | --- | --- | --- |
| pK7-nIEP  pK7- NLSm  pK7-nYYAA  pK7-nYAHH  pK7-nKA | GFP:IEP | n-IEPattB1 | 1-21 | 5’-GGGGACAAGTTTGTACAAAAAAGCAGGCTTC ATGACTTCGGAAAGTACGACA-3’ |
|  |  | n-IEPattB2 | 1260-1240 | 5’-GGGGACCACTTTGTACAAGAAAGCTGGGTC  TCAGGTAAACGTGTTCGTTCC-3’ |
| pK7-cIEP | IEP:GFP | c-IEPattB1 | 1-21 | 5’-GGGGACAAGTTTGTACAAAAAAGCAGGCTTCACC ATGACTTCGGAAAGTACGACA-3’ |
|  |  | c-IEPattB2 | 1257-1237 | 5’-GGGGACCACTTTGTACAAGAAAGCTGG GTCGGT  AAACGTGTTCGTTCCGAA-3’ |
| pK7-nMat | GFP:Mat | n-MattB1 | 805-824 | 5’-GGGGACAAGTTTGTACAAAAAAGCAGGC TTCTACTGCAAGGATCAACGGCG-3’ |
|  |  | n-MattB2 | 1260-1238 | 5’-GGGGACCACTTTGTACAAGAAAGCTGG GTCTCAGGTAAACGTGTTCGTTCCGA-3’ |
| pK7-nRT | GFP-RT | n-RTattB1 | 136-155 | 5’-GGGGACAAGTTTGTACAAAAAAGCAGGCTTC CTTGCAGCAAACCTCTACAA-3’ |
|  |  | n-RTattB2 | 917-896 | 5’-GGGGACCACTTTGTACAAGAAAGCTGG GTCTCAGAAGAACTCGTCCCGCTGTG-3’ |
| pK7-n4 | GFP:IEPsac | n-IEPattB1 | 1-21 | 5’-GGGGACAAGTTTGTACAAAAAAGCAGGCTTC ATGACTTCGGAAAGTACGACA-3’ |
|  |  | n-IEPsacattB2 | 1260-1247 | 5’-GGGGACCACTTTGTACAAGAAAGCTGG  GTCTCAGGTAAACGTGTTCGTTCC-3’ |
| pK7-nΔC29 | GFP:ΔC29 | n-IEPattB1 | 1-21 | 5’-GGGGACAAGTTTGTACAAAAAAGCAGGCTTC ATGACTTCGGAAAGTACGACA-3’ |
|  |  | n-ΔC29attB2 | 1170-1150 | 5’-GGGGACCACTTTGTACAAGAAAGCTGG  GTCTCATGTCTTATGGGACTGAAAGCG-3’ |

**Table S1: Primer pairs used to generate the IEP PCR fragments for recombination with pDONR^TM^221.**
